# Photothermal Effect of Modulating Laser Irradiation on the Thermal Diffusivity of Al_2_O_3_ Nanofluids

**DOI:** 10.1186/s11671-019-2869-2

**Published:** 2019-01-28

**Authors:** Monir Noroozi, Bijan Mohammadi, Shahidan Radiman, Azmi Zakaria, Raba’ah Syahidah Azis

**Affiliations:** 1 School of Mechanical Engineering, Iran University of Science and Technology, Narmak, Tehran, 16846 Iran; 2 School of Applied Physics, Faculty of Science and Technology, Universiti Kebangsaan Malaysia, 43600 Bangi, Selangor Malaysia; 3 Department of Physics, Faculty of Science, Universiti Putra Malaysia, 43400 Serdang, Selangor Malaysia; 4 Institute of Advanced Materials, Universiti Putra Malaysia, 43400 Serdang, Selangor Malaysia

**Keywords:** Photothermal effect, Continuous wave laser, Fragmentation of nanoparticles, Thermal diffusivity of nanofluids

## Abstract

Modulated continuous wave (CW) lasers cause photothermal effect that leads to rapid optical absorption and generation of thermal waves around the irradiated nanostructures. In this work, we examined the effect of modulated CW laser irradiation on the particle fragmentation process to enhance the thermal diffusivity of nanofluids. A facile and cost-effective diode laser was applied to reduce the agglomerated size of Al_2_O_3_ nanoparticles in deionized water. The thermal wave generation, which was determined by the modulated frequency of the laser beam and the optical and thermal properties of the nanofluid, is also briefly discussed and summarized. The influence of laser irradiation time on nanoparticle sizes and their size distribution was determined by dynamic light scattering and transmission electron microscopy. The thermal diffusivity of the nanofluid was measured using the photopyroelectric method. The data obtained showed that the modulated laser irradiation caused the partial fragmentation of some agglomerated particles in the colloids, with an average diameter close to the original particle size, as indicated by a narrow distribution size. The reduction in the agglomerated size of the particles also resulted in an enhancement of the thermal diffusivity values, from 1.444 × 10^−3^ to 1.498 × 10^−3^ cm^2^/s in 0 to 30 min of irradiation time. This work brings new possibilities and insight into the fragmentation of agglomerated nanomaterials based on the photothermal study.

## Background

Metal oxide nanofluids have attracted a lot of attention due to their enhanced thermal properties which allows them to play specific roles in the development of heat transfer equipment. Metal oxides nanofluids is well known to possess enhanced thermo-physical properties such as thermal diffusivity, thermal conductivity, and convective heat transfer coefficients compared to those of base fluids like oil or water. Al_2_O_3_ is an interesting oxide, as a material for enhancing the heat transfer, because of its high thermal conductivity. The thermal conductivity of nanofluids act as important properties in developing an energy-efficient heat transfer equipment, mainly used in industrial field such as automotive, electronics equipment, and medical applications. The thermal properties of nanofluids are sensitive to the size and shape of the nanoparticles (NPs) and their base fluids [1–5]. This poses a problem as NPs have a tendency to aggregate quickly and causes a decrease in thermal properties of the nanofluids [6–8]. Recently, laser-produced nanoparticles methods have been used to modify and generate NPs directly in the base fluids [8–10] to be used in chemical, optical and thermal engineering, phototherapy, catalysis, and heat transfer. The size and dispersion of it can be controlled by varying laser parameters, such as the laser wavelength, pulse duration, number of laser pulses, and pulse energy [11, 12]. In general, the interaction between the laser and the particles not only caused photothermal ablation but also generated thermal waves (TWs) around the nanostructures, and their surrounding medium, which lead to a reduction in size of the particles or the formation of NPs with a specific size distribution. Studies on the optical fabrication of NPs by laser irradiation showed that the laser ablation of solid targets [12–15] and fragmentation from suspended microcrystalline powders [16–26] can be employed by either using powerful pulsed lasers or low-power intensity CW laser sources. Pulsed lasers have been used in many studies for the laser ablation of solid targets in liquids. Although laser irradiation is a useful technique to assist the formation of NPs in nanofluids, the efficiency of the laser irradiation process is quite sensitive to the pulse duration. However, in the case of pulsed laser irradiation, the NP size and distribution were significantly influenced by the number and irradiation times of the laser pulses. This implies that it was still difficult to achieve more particles production with control over the size distributions of the produced nanoclusters. In recent years, CW lasers have been used in several studies for the fabrication of NPs [27–30]. There are several advantages in using CW laser sources as opposed to other optical sources, as they are generally less expensive, smaller, and have a more portable setup that can be potentially combined with other devices, especially as a photothermal therapy source for medical application and the reshaping and fabrication of nanomaterials [30, 31]. Recently, many experimental and theoretical investigations aimed at understanding the mechanism of laser irradiation have been performed [24, 31–36]. On the basis of calculations and experimental confirmations, the laser ablation and fragmentation of NPs can be driven by the photothermal (PT) effect [37–41]. The PT effect allows for the optimization and monitoring of the efficiency of the laser irradiation with different optical sources in different experimental designs [42–49]. Modulated CW laser is generally used in applications involving the PT effect. It can be a good PT source of light given an optimal modulation frequency. An increase in the efficiency of the thermal waves and the signal to noise ratio (S/N) can be observed, making it more suitable for the NPs fragmentation process. Moreover, a careful optimization of the experimental conditions can establish control over size distributions of the produced nanoclusters and thermal properties of nanofluids. However, no detailed study exists in literature for the PT effect of modulating CW laser on the formation and size of NPs and their thermal properties.

In the paper, a CW diode laser was used for the fragmentation of clustered Al_2_O_3_ particles to enhance the thermal diffusivity of the nanofluids, under various irradiation times. The basis of the thermal wave generation of the modulated CW laser beam was briefly summarized and the effect of the modulated beam frequency and physical parameters were discussed. The results of the laser fragmentation process were analyzed using transmission electron microscopy (TEM) and dynamic light scattering (DLS) analysis. Finally, the effect of laser treatment on the thermal diffusivity of the nanofluids was investigated. The photopyroelectric (PPE) technique was used as a valid method for measuring the thermal diffusivity of the nanofluids with very high precision and resolution.

### Thermal Wave Generation of the Modulated Laser Beam

**Fig. 1:**
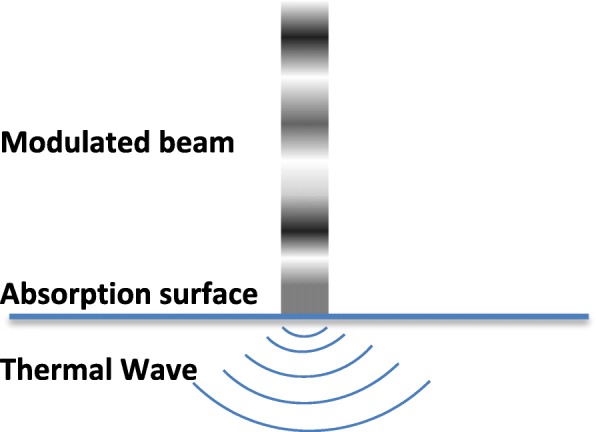
Photothermal phenomena caused by illumination of a surface by modulated beam of the light

In the CW modulated laser, the absorption of the modulated incident light beam causes a thermal wave field, which is a result of the periodic temperature distribution on the surface [50]. In the case of modulation with different frequencies, when the surface of an absorbing material is irradiated with a modulated optical radiation at frequency *f*, where flux is the source intensity and is the modulated angular frequency of the incident light, the absorption of the modulated incident light beam will results in the generation of thermal waves on the sample surface. Figure 1 is a schematic illustration of the phenomena resulting from the exposure of a sample surface to a modulated CW laser beam. The acoustic thermal energy that arises due to the PT effects leads to the transport of thermal waves through the sample and surrounding medium.

In the case of nanofluid with an amount of solid particles, thermal waves generated in the solid particles diffuse into both media including the other solid particles and the adjacent fluid layer, in a 3-D thermal wave field. The thermal wave diffuses in 3-D, if the heat source is small compared to the lateral dimensions of the sample; this thermal diffusion equation needs to be solved using cylindrical symmetry. Based on Fourier series theory, the relationship between the temperature gradient (∇*T*) and the conduction rate (*k*) in the direction of energy flow (*q*) in a material is

1 $$ q=-k\nabla T $$,

and the differential equation of heat conduction is [50]

2 $$ {\nabla}^2T=\frac{1}{\alpha}\frac{\partial T}{\partial t} $$

The thermal diffusion equation in the solid particle, as a distributed heat source, is [51]

3 $$ \frac{\partial^2{T}_s}{\partial {r}^2}+\frac{1}{r}\frac{\partial {T}_s}{\partial r}+\frac{\partial^2{T}_s}{\partial {z}^2}=\frac{1}{\alpha_s}\frac{\partial {T}_s}{\partial t}-\frac{1}{2k}{I}_0\left(1+{\mathrm{e}}^{i\omega t}\right) $$

The thermal diffusion equations in base fluid medium can be written as [51]

4 $$ \frac{\partial^2{T}_l}{\partial {r}^2}+\frac{1}{r}\frac{\partial {T}_l}{\partial r}+\frac{\partial^2{T}_l}{\partial {z}^2}=\frac{1}{\alpha_l}\frac{\partial {T}_l}{\partial t} $$

**Fig. 2:**
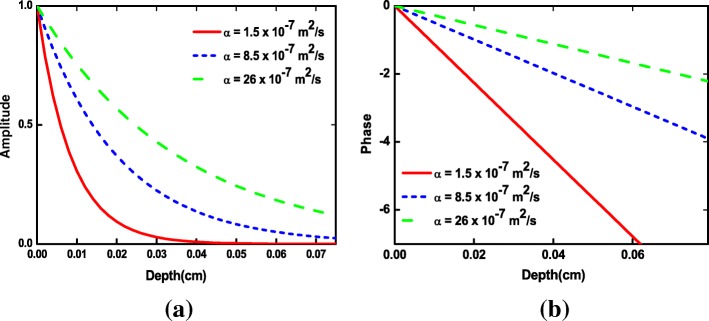
**a** Amplitude and **b** phase of Eq. (5) with thermal diffusivity *α* as a parameter

The thermal wave propagation in a material depends on its thermal diffusivity *α* = (*k*/*ρc*)^1/2^, where *k* denotes the thermal conductivity, *ρ* the density, and *c* the heat capacity. The thermal wave propagating *T*(*x*,*t*) in the one-dimensional approach can be found by solving the complex equation

5 $$ T\left(x,t\right)={T}_0{e}^{\left(-x/\mu \right)}{e}^{\left[i\left(\omega t-x/\mu \right)\right]} $$

where *σ*_*j*_ = (1 + *i*)/*μ*_*j*_ is thermal wave diffusion coefficient, *μ* = (*α*/π*f*)^1/2^ is the thermal diffusion length at frequency *f*, and α is the thermal diffusivity of liquid sample; *T*_*o*_ is the initial change in temperature produced by the source, and the wave is attenuated by a factor of 1/*e*. Figure 2a, b clearly shows the thermal decay of the amplitude and phase of the thermal waves (Eq. 5) as a function of distance (depth) away from the source at *x* = *x*_0_. The rate of the steep (exponential) amplitude decay away from the source depends on the thermal diffusivity of medium; the higher the diffusivity, the gentler the slope. A similar behavior is observed for the phase. For the low thermal diffusivity, the induced thermal waves have a short thermal wavelength and they are subjected to a large attenuation. Therefore, heat transfer at the particle surface does not occur, and PT effect starts to reduce, because the main characteristic of thermal wave is that it decays strongly [52, 53]. This simulation showed that the thermal effect is predominated at particles with high thermal diffusivity and induced peeling off of the particle surface. In this work, water is used as liquid of higher thermal diffusivity than of the other liquids, thus produces higher S/N compared to the latter.

## Methods

### Preparation of Nanofluids

The nanofluids were prepared by dispersing 0.05 g Al_2_O_3_ NPs (11 nm, Nanostructured and Amorphous Materials, Inc.) into 25 ml deionized (DI) water. One volume percent polyvinylpyrrolidone (PVP) (K25, MW–29000, Aldrich Chemistry) was added to stabilize the nanofluids; Al_2_O_3_ NPs in water have a strong tendency to form aggregates [54, 55]. The suspension was stirred in about 1 h then the mixture was subjected to probe sonication for 30 min (VCX 500, 25 kHz, 500 W) to ensure homogeneous particle distribution. After the suspension was mixed thoroughly for 30 min, the hydrodynamic size of the agglomerated particles in the solution was monitored using DLS.

### Laser Fragmentation Process

**Fig. 3:**
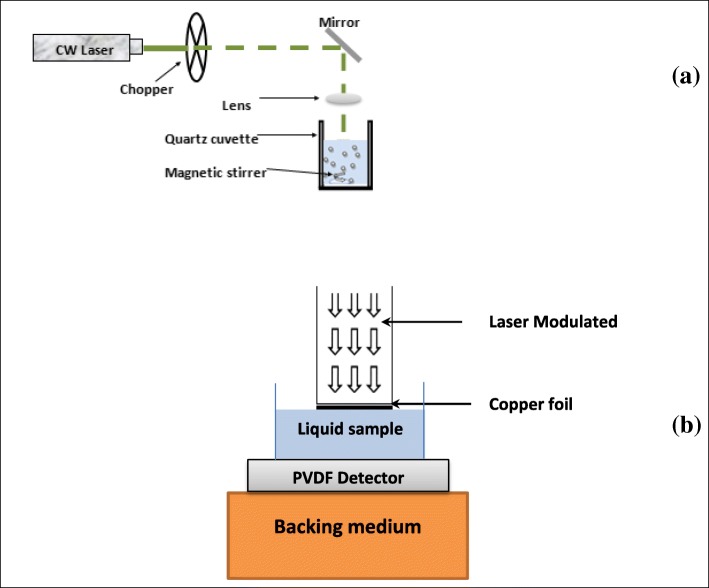
**a** Block diagram of experimental setup used in fragmentation of particles by a modulated CW laser beam and **b** schematic view of the photopyroelectric (PPE) configuration detection cell for thermal diffusivity measurement

The laser fragmentation process by a modulated CW laser beam is depicted in Fig. 3a. The experimental setup for the CW modulated laser is a fairly simple experiment. A cuvette containing 2 ml of the sample solution was placed on a stirring plate and irradiated along the vertical axis with a CW diode pumped solid state laser (532 nm, 200 mW, MGL 150(10)). The laser was modulated using an optical chopper (SR540) at a modulation frequency of 10 Hz, to produce a reasonably high S/N. The laser was focused on about 0.1 mm (2.5 kW/cm^2^) of the solution surface in the quartz cuvette using a 10 cm focal length lens. Magnetic stirring was carried out in order to ensure homogeneous particle distribution. The process was repeated in 10 and 30 min. After each experiment, the morphologies of the obtained colloidal suspensions were analyzed by TEM (H-7100, Hitachi, Tokyo, Japan), and size distribution of the Al_2_O_3_ NPs in solution were determined using the UTHSCSA ImageTool (version 3.0) software. The hydrodynamic size of the agglomerated particles in the solution was obtained from the DLS analysis using the Nanophox Analyzer (Sympatec GmbH, D-38678), and an average was taken from at least four measurements.

### Thermal Diffusivity Measurements

The details of the experimental setup for thermal diffusivity measurements in liquid samples can be found elsewhere [56]. The PPE technique has been shown to be a useful method to analyze thermal properties of several kinds of liquids, with very high precision and resolution [51–53, 56–59]. The advantage of this technique is that we used a small limited volume together with a short measurement time [56–59]. PPE technique was employed to measure the thermal diffusivity of the Al_2_O_3_ nanofluids. Figure 3b shows the PE signal generation chamber or cell utilized in the PPE technique. The cell contained a copper foil (50 μm thickness) acted as a PE generator and a 52-μm polyvinylidene difluoride (PVDF) film (MSI DT1-028 K/L) acted as a PE detector, and the nanofluid sample was placed in this cavity. Since PVDF film is very flexible, it was fixed with silicon glue to Perspex substrate. The copper foil surface was coated with a very thin layer of carbon soot to act as an efficient light-to-heat converter. The intensity of a diode laser (532 nm, 200 mW) was modulated by the optical chopper (SR540) before illumination on copper foil. In the cell, the thermal wave propagates across the liquid and reaches the PE detector, which generates a PE signal proportional to the intensity of the thermal wave. The PE signal generated by PVDF detector was analyzed by using a lock-in amplifier (SR.530) to produce PE amplitude and phase signals. To avoid vibrations and possible contributions of the PVDF sensor, its bottom rear face was attached to a Perspex container. The experiment was done for the cavity scan. The frequency at 6.7 Hz was chosen for a thermally thick regime for reasonably high signal amplitude in the system. The measurements were performed at room temperature (approximately 22 °C). Measurements were repeated five times for a particular sample, and the averaged thermal diffusivity value was taken. The LabVIEW software, installed in PC, was used to capture the PE signal and the data were analyzed using Origin 8. The temperature field of the experimental system can be calculated according to the thermal wave cavity conduction theory [57]. The PE signal detected by PVDF sensor, the PE signal (*V*), is determined by the cavity length distance and sample thermal diffusivity:

6 $$ V\left(f,l\right)={V}_0\exp \left(-\left(1+i\right) AL\right) $$

7 $$ \ln \left|V\left(f,l\right)\right|=\ln \left|{V}_0\right|- AL $$

8 $$ \varphi ={\varphi}_0- AL $$

where *A* = (*πf*/*α*)^1/2^ to obtain this expression, *V(f*, *l)* is the complex PE signal, *V*_*o*_ and *φ* are the amplitude and phase of PE signal, *f* is the modulation frequency, and *α* is the thermal diffusivity of sample. From the slope fitting parameter *A* = (*πf*/*α*)^1/2^ of phase and ln(amplitude) as a function of cavity scan, thermal diffusivity of liquid can be calculated [58].

## Results and Discussion

### Thermal Wave Enhancement

There are some key parameters that should be considered to generate strong thermal wave amplitude:

- Modulation frequency of the modulation light

From Eq. (5), there should be an optimum modulation frequency to maximize the thermal wave amplitude. Unlike other waves, thermal wave is very heavily damped with a decay constant equal to the thermal diffusion length of the medium of propagation [52]. The thermal waves originating from no deeper than the thermal diffusion length in the material contribute to the heat propagation [53]. The thermal waves are reflected and transmitted at the interface and the amplitude of the thermal waves is attenuated within one thermal diffusion length of the sample. With increasing modulation frequency according to Eq. (5), the thermal diffusion length decreases, and only light absorbed within the surface layer contributes to the signal, while the thermal waves will propagate deep into a solid if the material has a high thermal diffusivity or if the thermal wave frequency is low. In the experiment, one should carefully choose the modulation frequency in order to get a sharp resonant peak (actually a trough). The modulation frequency is chosen in the spatial range. If the frequency is too low, the signal is strong, but the peak is too flat for precise determination of its maximum. While if the frequency is too high, the peak is quite sharp, but the signal-to-noise (S/N) ratio is compromised, which makes identification of the peak position difficult.

**Fig. 4:**
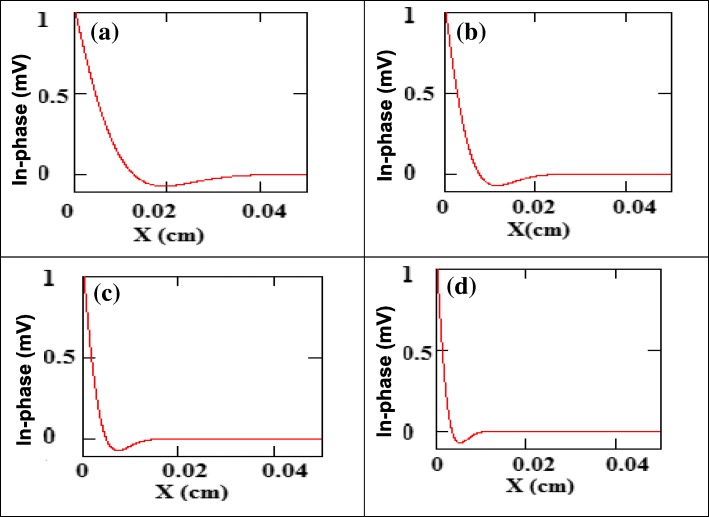
The real (in-phase) part of PE signal vs relative cavity length for water at different frequencies: **a** 7 Hz, **b** 20 Hz, **c** 50 H, and **d** 100 Hz, thermal diffusivity of water (*α*_*w*_,=0.00145 cm^2^.s^−1^)

Figure 4 shows the simulated real (in-phase) part of PE signal as a function of cavity length of water, at different frequency from 7 Hz to 100 Hz. It can be seen that the S/N ratio was higher for lower frequencies, 7 Hz, while the peak was too flat for a precise determination of its maximum (Fig. 4a). However, the peak was quite sharp at higher frequencies, 100 Hz, (Fig. 4d), with a smaller output signal was obtained, which made the identification of the peak position difficult [52]. It was experimentally found that with 10 Hz as the operating frequency, the S/N ratio was good in a range of frequencies and had satisfactory signal amplitude in the system.

b. Optical absorption of the nanofluids

Each particle is a light scattering and absorbing light object. The absorbed energy can be converted into heat, and the summation of the light absorption of the particles is thermal extinction. The thermal wave amplitude can be increased by increasing the optical absorption [52, 59] with in the nanofluids. Particle size, shape, and volume fraction, as well as the alternation of the base fluids, have a major effect on the optical absorption of the nanofluids. The Al_2_O_3_/water nanofluid had favorable optical absorption. The optical energy absorbing 13% of water increased with Al_2_O_3_ NPs in the base fluid and was further enhanced when the concentration of the NPs increased. With a high concentration of NPs, the incident light of every particle was absorbed in a thin surface layer.

c. Specific heat capacity of the nanofluids

Fabrication of small particle size Al_2_O_3_ in solution by using a modulated CW laser fragmentation can increase the heat storage of the nanofluid, due to the fact that the specific heat capacity of base fluid decreased with decreasing the particle size and increasing amount of NPs, due to increasing the surface area-to-volume ratio of the particles [6]. Therefore, the smaller specific heat capacity of the nanofluid allowed thermal wave amplitude due to enhanced temperature rise and heat transfer.

d. Thermal diffusivity of the nanofluids

Heat is transferred from the solid particles to the surrounding medium followed by thermal wave expansion, where the amplitude of the thermal waves (TWs) is a strong function of the thermal diffusivity. As shown in Fig. 2, a larger thermal diffusivity is usually preferred for higher thermal diffusion lengths and the thermal wave amplitude below the surface decays slowly. Therefore, the large thermal diffusivity of the base fluid is crucial for effective heat transfer from the solid particles to the fluid, thus, maximizing thermal wave generation. In this work, water with a high thermal diffusivity (0.00145 cm^2^/s) was a good base fluid for efficient thermal wave generation. The thermal diffusivity of water increased with an increasing amount of NPs, due to increasing Brownian motions [56]. The higher thermal diffusivity and smaller specific heat of the Al_2_O_3_ nanofluid compared to water allowed it to be excellent thermal wave generator.

## Experimental Results

### Laser Fragmentation of the Al_2_O_3_ Nanoparticles

**Fig. 5:**
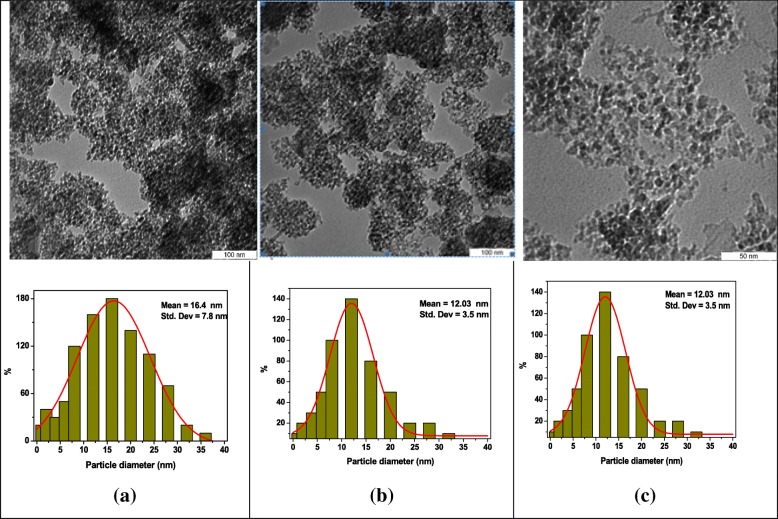
The TEM images and the relative size histograms of the Al_2_O_3_-NPs **a** before (16.4 ± 7.8 nm) and after laser irradiation, at **b** 10 min (14.2 ± 5.4 nm), and at **c** 30 min (12.03 ± 3.5 nm), respectively

**Fig. 6:**
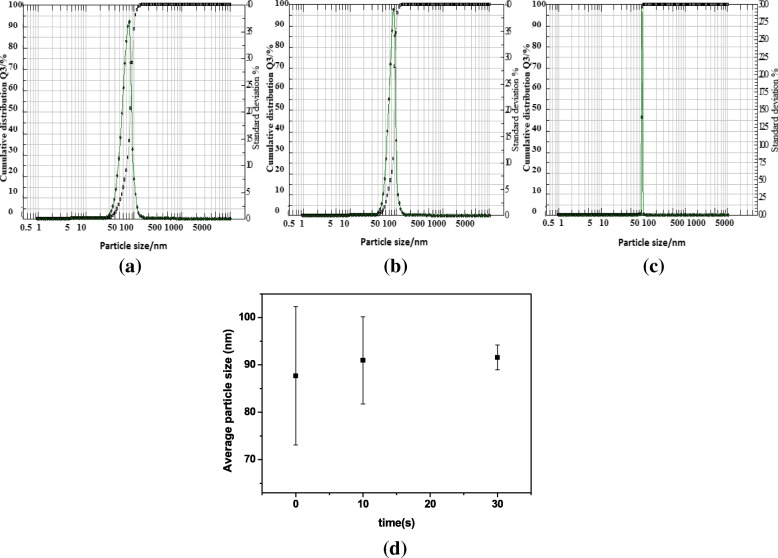
The distribution density determined using the Nanophox analyzer of Al_2_O_3_ particles in the suspensions **a** without, with irradiation after **b** 10 min and **c** 30 min, and **d** hydrodynamic diameter distribution of NPs in nanofluids as a function of irradiation times

The TEM images showing the average size and size distribution of the Al_2_O_3_ NPs in deionized water/PVP solution before and after 10 min and 30 min of irradiation are shown in Fig. 6. It can be seen that the collected material was composed of clusters of nearly spherically shaped particles, dispersed in a highly porous material. Some agglomeration of around 100 nm in diameter was observed and the mean size of the Al_2_O_3_ NPs was about 16.4 ± 7.8 nm (Fig. 5a). The porous material range was reduced and the mean particle size was found to be 14.2 ± 5.4 nm after 10 min of irradiation (Fig. 5b). Figure 5c showed that the Al_2_O_3_ NPs were almost uniformly distributed and narrow in size (12.03 ± 3.5 nm) after 30 min of irradiation as a result of the absorption of laser energy which lead to the fragmentation of the particles [25]. However, the fragmentation rate of the particles decreased when the NPs reached their critical size after 30 min of irradiation. Increasing the total number of particles resulted in an increase in the NPs concentration, and the agglomeration of these small particles hence the light absorption of particles in solution was decreased. The data obtained showed that the effect of laser irradiation on the distribution size was more than on the size of particles [11].

The hydrodynamic diameter of the Al_2_O_3_ particles in the nanofluids can provide information on the stability of the nanofluids. Figure 6 shows the distribution density function of the NPs in the suspension (a) without and with irradiation after (b) 10 min and (c) 30 min. The gravity of the density curve provides the mean sphere diameter. In addition, a narrow hydrodynamic size of the particles was obtained when laser exposure after 10 and 30 min (b and c), while the particles before irradiation had a widely spread interface indicating a greater degree of polydispersity (Fig. 6a). The data obtained showed that a sharper distribution curve of the highly homogeneous particles was obtained after laser irradiation. This could be due to the fragmentation of the particles after laser irradiation. Longer laser irradiation times resulted in a higher fragmentation of the particles and hence higher number of particles in solution with a sharp distribution. It was observed that the tendency to agglomerate increased with an increase in the number of smaller particles in the water [7, 54, 55]. Figure 6d shows the hydrodynamic diameter distribution of the Al_2_O_3_ particles in the nanofluids with diameters of 87.7 ± 14.59 nm, and 90.97 ± 9.21 nm and 91.57±2.61 nm for before and after 10 and 30 min of irradiation, respectively. It was found that the size distribution of the particles decreased from ~ 15 to ~ 3 nm, when the irradiation times increased from 0 to 30 min, respectively. The fragmentation of the agglomerates took place via a direct absorption of the laser with an end result of particles that were almost uniform in size distribution as seen from the Nanophox and TEM data. The data obtained showed that the effect of laser irradiation on the distribution size was more than that on the size of particles. However, the hydrodynamic size of the NPs obtained from the Nanophox analyzer was always larger than the size of the dry particles obtained from TEM as the hydrodynamic average diameter is the size of agglomerated particles in solution. The sharp distribution and size reduction effects observed here have been reported in the literature [7–10, 16–23].

### Thermal Diffusivity Measurements

**Fig. 7:**
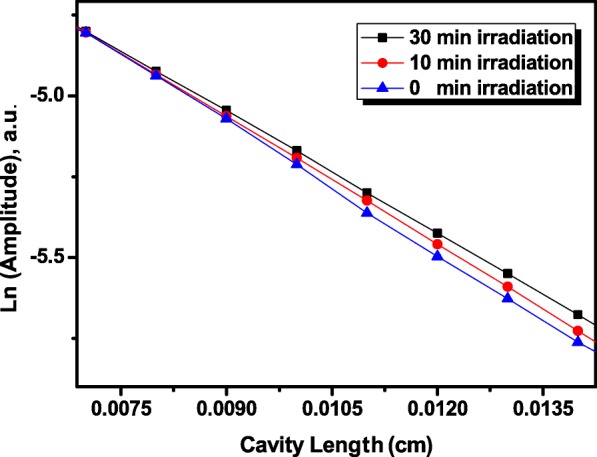
Typical logarithmic amplitude as a function of the relative cavity length of Al_2_O_3_ nanofluids at different irradiation time [0, 10, and 30 min]

In order to measure the effect of laser irradiation on the thermal diffusivity of the nanofluids, firstly, the experimental setup was calibrated using distilled water as a standard liquid. The thermal diffusivity was measured from fitting the PE signal of the ln(amplitude) (Eq. (7)) and phase (Eq. (8)) versus the cavity length. The average for distilled water was (1.4460.011) × 10^−3^ cm^2^/s, which differed by < 1% from the literature [56]. Figure 7 shows the linear plots of logarithmic amplitude versus the cavity length of the Al_2_O_3_ nanofluids at different laser irradiation time from 0 to 30 min as a function of the relative cavity length. The slopes of the PE signal (ln (amplitude), phase, and average) and the resulting thermal diffusivity values measured in the present work are summarized in Table 1.

**Table 1:** Summarized results for thermal properties of Al_2_O_3_ nanofluids at different laser irradiation times

Time (minutes)	*A*_phase_	*A*_amplitude_	*A*_average_	*α*(10^−3^ cm^2^/s)
0	120.6 ± 0.8	122.6 ± 0.6	121.6 ± 0.7	1.444 ± 0.008
10	119.4 ± 0.6	121.8 ± 1.0	120.6 ± 0.8	1.468 ± 0.011
30	118.4 ± 0.9	120.4 ± 0.6	119.4 ± 0.7	1.498 ± 0.009

The thermal diffusivity showed an enhancement compared to the base fluid. However, for the nanofluid without irradiation, the thermal diffusivity was (1.444 ± 0.008) × 10^−3^ cm^2^/s, which was lower than base fluid. This could be due to the low thermal diffusivity of PVP in the nanofluids. The thermal diffusivity gradually increased around 3–6% after laser irradiation, which was defined as an aging effect [56, 57]. The increase in the thermal diffusivity with longer irradiation time was a consequence of the decrease in the clusters and agglomerate sizes, due to the fragmentation of the larger NPs [7–10]. Generally, the density of the number of particles or volume fractions of the particles increased and it was evident that the particle size reduction increased the nanoscale mixing effects, such as Brownian motions [56]. Therefore, this could help to enhance the thermal diffusivity of the nanofluids. However, the increase in the number of particles in the solution had an influence on the rate of laser fragmentation, due to the attenuation of laser light in the liquid at high concentrations.

In principle, the interaction between the CW laser beam (in our experiment 10^3^ W/cm^2^) and the Al_2_O_3_ clusters is governed by thermal effects which depends on the characteristics of the laser radiation and the nature of the particle. Hence, considerable research has been directed towards decreasing the size of the particles using various nanosecond (ns) and femtosecond (fs) lasers running at different pulse duration [13–19, 21, 25–27]. Coincidentally, the exact same result was obtained through our experiments. As a result of the nanofluids, in the laser irradiation, time affected mainly the particles rather than their size. This was probably because of the effect of the laser irradiation on the fragmentation of the agglomerated particles to the smaller NPs thus increasing the homogeneous particle distribution of the Al_2_O_3_ nanofluids. These results demonstrated the surprisingly narrow distributions, with size dispersions in the order of the mean size, which was confirmed by measuring TEM and Nanophox results. This suggested that the NPs were excited and heated by irradiation of the modulated CW laser with some heat loss to the surrounding water, while the absorption of the laser energy by the particles could cause further fragmentation of the particles to smaller possible sizes thus increasing the total number of particles in the solution [28]. In addition, the distribution of particle also decreased with an increase in the laser irradiation time, which has been reported with other materials, such as metal [11, 13, 14, 17] and metal oxide [9, 10, 29].

## Conclusions

In conclusion, we confirmed that the modulated continuous wave laser can be used as a good photothermal light sources to generate the thermal waves for fragmentation of the clustered Al_2_O_3_ particles and enhancing the thermal diffusivity of the Al_2_O_3_ nanofluids. Modulated CW laser technique shows an enormous promise for accurate characterization of the particle size distribution of Al_2_O_3_ nanofluids. There are some controlled experiments to optimize the thermal wave generation efficiency, such as the size of the particles, modulation frequency, thermal properties of particles, and base fluid. The results showed that the effect of laser irradiation on the distribution size was more on the size of particles. The thermal diffusivity of the Al_2_O_3_ nanofluid increased to 3–6% with the increase of irradiation times, due to the fragmentation of the NPs which in turn increased the total number of particles in the solution. Therefore, from this work, it predicated that inexpensive and compact CW diode lasers can be successfully designed and employed for the fragmentation of NPs in nanofluids.

## Nomenclature

- *I*_*o*_ Source intensity
- *ω* Angular frequency of modulated light
- *f* Modulation Frequency
- ∇*T* Temperature gradient
- *q* Energy flow
- *e* thermal wave diffusion coefficient
- φ phase of PE signal
- *μ* Thermal Diffusion Length
- *k* Thermal Conductivity
- *α* Thermal Diffusivity

## Abbreviations

3-D: Three-dimensional
CW: Continuous wave
DW: Deionized water
NPs: Nanoparticles
PE: Pyroelectric
PVDF: Polyvinylidene difluoride
PVP: Polyvinylpyrrolidone
S/N: Signal-to-noise
V: Amplitude of PE signal
